# Synaptic and metabolic gene expression alterations in neurons that are recipients of proteopathic tau seeds

**DOI:** 10.1186/s40478-020-01049-7

**Published:** 2020-10-19

**Authors:** Marta Perez-Rando, Simon Dujardin, Rachel E. Bennett, Caitlin Commins, Tara Nibhanupudy, Bradley T. Hyman

**Affiliations:** 1 Alzheimer Research Unit, Department of Neurology, Massachusetts General Hospital, Building 114, Room 2009, Charlestown, MA 02129 USA; 2 Harvard Medical School, Boston, MA USA

**Keywords:** Alzheimer’s disease, Neurofibrillary tangles, Tau seeding, RNA expression

## Abstract

Recent studies suggest that misfolded tau molecules can be released, and taken up by adjacent neurons, propagating proteopathic seeds across neural systems. Yet critical to understanding whether tau propagation is relevant in pathophysiology of disease would be to learn if it alters neuronal properties. We utilized high resolution multi-color in situ hybridization technology, RNAScope, in a well-established tau transgenic animal, and found that a subset of neurons in the cortex do not appear to express the transgene, but do develop phospho-tau positive inclusions, consistent with having received tau seeds. Recipient neurons show decreases in their expression of synaptophysin, CAMKIIα, and mouse tau in both young and old animals. These results contrast with neurons that develop tau aggregates and also overexpress the transgene, which have few changes in expression of metabolic and synaptic markers. Taken together, these results strongly suggest that tau propagation impacts neuronal functional integrity.

**Electronic supplementary material:**

The online version of this article (10.1186/s40478-020-01049-7) contains supplementary material, which is available to authorized users.

## Introduction

Accumulating evidence suggest that hyperphosphorylated or misfolded conformers of the microtubule associated protein Tau, which accumulates as aggregates in several neurodegenerative diseases including Alzheimer’s disease (AD) and some forms of frontotemporal dementia, have the ability to “spread” through the brain [4, 5, 7, 12, 20, 23]. It has now been suggested that tau can form proteopathic seeds that contaminate neurons through a trans-synaptic mechanism in a prion-like manner [22, 31]. Consistent with this idea, tau was shown to actively spread from neuron to neuron in various model systems. For example, in a transgenic model where tau is exclusively expressed in the entorhinal cortex, tau spreads to limbic and neocortical structures [7, 21]. In addition, after focal injections of viral vectors, human tau spreads from the viruses injection site to different connected areas [15, 32]. Additionally, direct injections of human tau aggregates into the brain of mice can lead to aggregate formation in neurons [3, 11, 19]. In these models, neurons that receive projections from tau expressing neurons develop tau aggregates suggesting the existence of a mechanism of proteopathic seeding happening in tau propagation “recipient” neurons [25]. However, despite detailed studies of the mechanisms of tau propagation, it is unknown how tau uptake impacts the biology of the recipient neurons.

Alterations in gene expression in tangle bearing neurons have been described in AD brain tissue, where some neuronal genes are specifically downregulated [9]. Among these, genes related to synaptic transmission and metabolism significantly decrease their expression [8, 9, 27]. Canchi et al. showed a differential expression in AD patients of different subunits of the RNA polymerase II (RNA Pol II), synaptophysin (SYP) and CaMKII. Specifically, SYP expression is decreased in tangle bearing neurons of AD patients when compared to other pyramidal neurons [8]. Here, we explore whether these differences are recapitulated in a well-described mouse model of tauopathy, the rTg4510 strain, which overexpresses a mutant P301L form of human tau, and that develops tau aggregates in cortical and hippocampal neurons as the animals age [24, 26]. We took advantage of a new observation that these mice are in some sense chimeras- there is a modestly large neuronal subpopulation in which the transgene is not expressed. Thus we could find (1) neurons expressing the transgene but without evidence of AT8 positive tau aggregation; (2) neurons in which the transgene is expressed and AT8 positive tau aggregation occurs; (3) neurons that do not express appreciable amounts of the transgene, but which, nonetheless, contain AT8 positive tau aggregates; and (4) neurons that do not express the transgene and do not contain AT8 positive aggregates. We examined two independent cohorts of mice, one younger and one older, to assess reproducibility and also assess potential age effects. This allowed us to address the question of whether the presence of the transgene (and hence, soluble tau), and/or aggregated tau, impacts the phenotypes of synaptic and metabolic dysfunction in neurons that are the recipients of proteopathic seeds.

## Materials and methods

### Animals

All animal experiments were performed in accordance with the Massachusetts General Hospital’s and McLaughlin Research Institute’s Institutional Animal Care and Use Committees. Mice were housed under a 12-h light/dark cycle and were given food and water ad libitum.

For these experiments we used both male and female mice from the tetracycline-responsive element strain FVB-Tg(tetO-MAPT*P301L)4510/Kha/Jlws (rTg4510). Five 12-month-old mice were used to study gene expression on NFT-bearing neurons of old animals, whereas seven 4 to 6-month-old mice were used to study these same traits on young animals. For the control assay to ensure all cell types were accurately represented using our method, we used three 4–6 month old rTg4510s placed on a doxycycline diet to prevent the expression of HsMAPT (200 mg/kg DOX, Fisher Scientific) ad libitum to suppress the CamkIIα-tTA-driven human tau transgene expression.

At 4–6 months old (young animal group) and 12 months old (old animal group), mice were deeply anesthetized with isoflurane and were euthanized by cardiac puncture. All animals were quickly perfused with chilled phosphate-buffered saline and brains were removed. The right hemisphere was then incubated in 4% paraformaldehyde (Electron Microscopy Sciences, #50-276-33) for 24 h at 4 °C, and then equilibrated in 30% sucrose in phosphate-buffered saline (PBS). Sections (40 µm thick) were sliced using a freezing microtome and were collected at 400-µm intervals.

### Fluorescence in situ hybridization assay

We performed the RNAScope protocol following the manufacturer instructions for fixed-frozen tissue. Briefly, the slides were first dehydrated and then exposed to RNAScope Hydrogen Peroxide solution (ACD #322335) for 10 min. The samples were then immersed in RNAScope target retrieval reagents solution (ACD #322000) at 98–102 °C for 15 min, followed by a 30 min exposition to RNAScope Protease III (ACD #322337) at 40 °C using the HybEZ II hybridization system (ACD #PN 321710), a temperature that will be kept constant for all incubation steps during the entire assay.

A channel 2 probe against human MAPT (Hs-MAPT-Tg4510-C2, ACD #417491-C2) was diluted 1:50 in channel 1 probes for the Poly(A) tail (RNAScope probe-PolyA, ACD#318631), mouse MAPT (RNAScope probe-MmMAPT-no-X-Hs, ACD #417481), mouse CaMKIIα (RNAScope probe-Mm-Camk2a, ACD #445231), mouse SYP (RNAScope probe-MmSYP, ACD #426521) and mouse POLR2A (mandatory subunit of the RNA Pol II, RNAScope probe-MmPOLR2A, ACD #312471). These probe combinations were then added to the tissue, and placed in the HybEZ II oven at 40 °C for 2 h. Afterwards, consecutive amplification steps were performed in the same oven, incubating the samples with RNAScope Multiplex FL v2 AMP1 (ACD #323101) for 30 min., RNAScope Multiplex FL v2 AMP2 (ACD #323102) also for 30 min., and RNAScope Multiplex FL v2 AMP3 for 15 min (ACD #323103). The HRC-C1 signal was then developed incubating the samples with RNAScope Multiplex FL v2 HRP-C1 in the oven (ACD #323104), followed by a 30 min. incubation step with TSA Plus Cy5 (Akoya Biosciences, #NEL745E001KT). After this step, the developing HRP signal was stopped adding RNAScope Multiplex FL v2 HRP blocker (ACD #323107) to the samples and incubating them for 15 min. Then, the channel 2 signal was developed incubating the samples with RNAScope Multiplex FL v2 HRP-C2 (ACD #323105) and TSA Plus Cy3 (Akoya Biosciences, #NEL744E001KT), and the signal was again stopped using the RNAScope Multiplex FL v2 HRP blocker, following the same times and temperature settings as previously done with the channel 1.

### Immunohistochemistry after RNAScope assay

The samples were then processed for an immunohistochemistry assay using a monoclonal mouse Phospho-Tau (Ser202, Thr205) antibody (AT8, ThermoFisher Scientific #MN1020), and all the steps were performed at room temperature. In summary, the tissue was blocked in 10% NGS for 1 h, followed by overnight incubation with the AT8 antibody (1:500). We then incubated the samples in a A488-conjugate donkey anti-Mouse IgG antibody (1:200, ThermoFisher Scientific, #A-21202) for 2 h. Finally, we coverslipped the samples using mounting media containing DAPI (Fluoromount-G with DAPI; ThermoFisher #00-4959-52).

### Imaging of mRNA expression

Samples were imaged using the Olympus confocal laser scanning microscope Fluoroview FV3000 with a 40X objective and a 2.34X digital zoom. A total of 6 image stacks were taken per animal in the somatosensory cortex (3 in layers I-II and 3 in layers IV-V). 6 focal planes comprised each image stack, and were separated by a step size of 1 µm.

### Image analysis

A single focal plane was extracted in every image stack, corresponding to the one presenting a higher pixel intensity value in the human MAPT channel (typically the second focal plane of each stack). To simplify the analysis, we randomly set a grid of 300 px (39.85 μm) width on each picture, and only cells placed on the grid lines were recorded using circular ROIs with a fixed diameter of 110 px (14.61 μm). The ROI size was kept constant because all analyzed cells belonged to a homogeneous subset of pyramidal neurons. In case two cells were overlapped, we always chose the left or lower one as long as their ROIs did not include any part of the other neuron, otherwise both cells would be discarded from the analysis. Two populations were then separately studied: NFT-bearing (AT8 +) neurons, and the rest of the cells (AT8-).

These two sets of ROIs were analyzed using a customized ImageJ/FIJI (NIH) macro that calculated the percentage of binarized area for each channel, using an adaptive threshold that corresponded to the pixel value on a set percentage of the top of each histogram. Each probe displayed different signal-to-noise ratios. Therefore, we customized the histogram percentage values for each gene studied to prevent misrepresenting binarizations. As for the Poly(A) analysis, the threshold value was set on the top 5% of the histogram. Regarding the genes of interest, this value of the histogram was set on the top 4% for the human MAPT probe, the top 0.9% for the mouse MAPT probe, the top 1% for the mouse CaMKIIα probe, the top 3% for the mouse SYP probe and the top 0.5% for the mouse RNA Pol II probe.

Afterwards, the data obtained within each set of ROIs was subsequently split in two groups based on their human MAPT expression: human MAPT positive (HsMAPT +) and human MAPT negative (HsMAPT-) cells, resulting in 4 cell groups per animal. For the study of the effect an NFT has on the neuron, we compared AT8 + vs AT8- cells, and only those expressing human MAPT were taken into account, to discard data contamination by other cell populations. To study how being seeded affects gene expression of these cells, we compared HsMAPT + vs HsMAPT- cells only on AT8 + neurons. Likewise, to study SYP expression in AT8- cells, we only considered morphologically similar neurons that also expressed SYP.

**Fig. 1:**
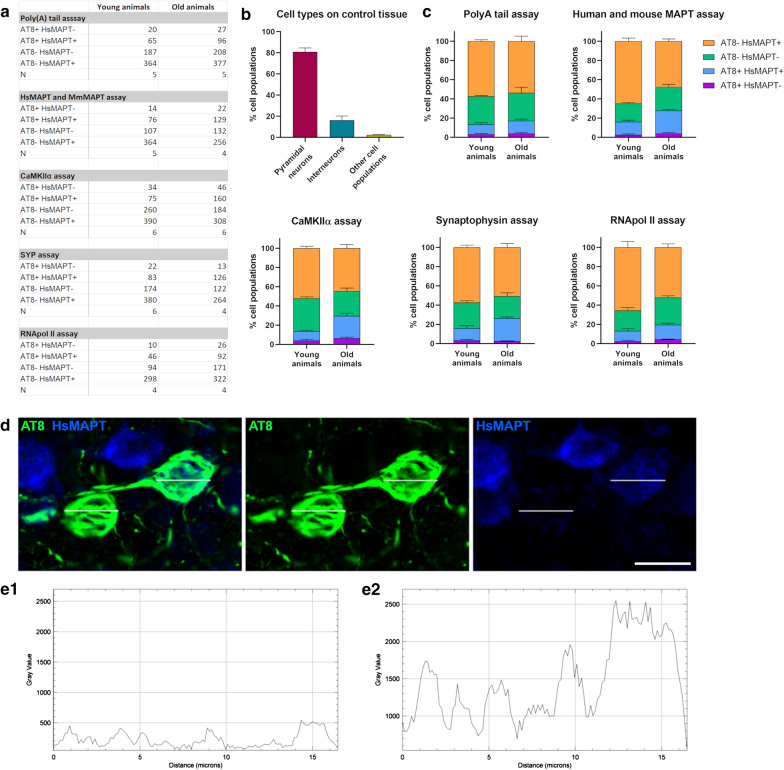
Cell populations analyzed in different RNAScope assays. **a** Table summarizing the total number analyzed for each cell population, and the number of animals, in every RNAScope assay in young and old animals. **b** Percentage of pyramidal neurons, interneurons and other cell types, performed in control animals and using the same method as the experimental RNAScope assays. **c** Percentages of each cell population in every experimental RNAScope assay, in young and old animals. **d** Representative images of a HsMAPT- (“recipient neuron”, left cell) and a HsMAPT + (right cell). **e** Consequent gray value histograms extracted from the HsMAPT channel shown in **d**. **e1** Histogram of the HsMAPT- (“recipient neuron”) and **e2** HsMAPT + neuron. Scale bar: 15 µm

To ensure the cell populations studied with our method were accurately representing the general proportions seen in the neocortex, we also performed the RNAScope assay using CaMKIIα and SYP probes in control animals (3 young rTg4510s mice injected with doxycycline). We followed the same steps used in the experimental RNAScope assays and calculated the percentage for each cell type (CaMKIIα + SYP + cells representing pyramidal neurons, CaMKIIα- SYP + cells representing interneurons, and CaMKIIα- SYP- representing other cell populations. See Fig. 1 for a summary of the cell populations analyzed in every assay, and a representative image and histograms of a neuron considered HsMAPT- (“recipient cell”) and HsMAPT + .

### Statistics

For all the statistical analysis we used GraphPad Prism v8.0.2 (Graphpad Software). In all the analyses we performed independent Student t-tests and the sampling unit was the animal. All p-values under 0.05 were considered as significant.

## Results

### The general transcript amount of tangle bearing neurons remains unaltered in both young and old animals

**Fig. 2:**
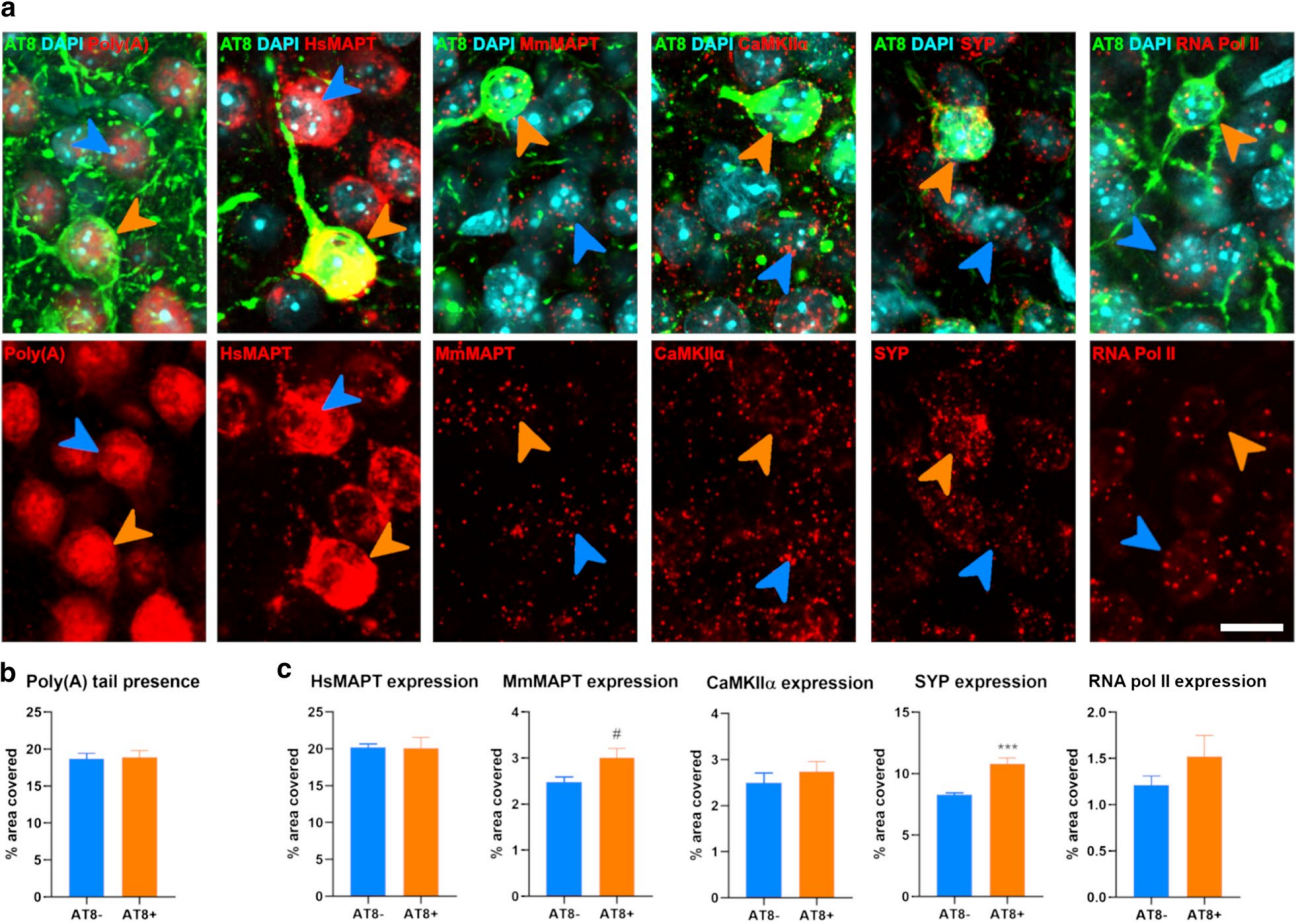
Transcript amount in NFT bearing neurons of young animals. **a** Single focal planes of neurons lacking (AT8-, blue arrowheads) or bearing NFT (AT8 + , orange arrowheads) for the total amount of mRNA (Poly(A) tail, left panel); and every gene analyzed. **b** Graphs summarizing the percentage of area covered with mRNAs (poly(A) tail). **c** Graphs summarizing the percentage of area covered with each probe targeting the genes of interest. Scale bar: 10 µm

**Fig. 3:**
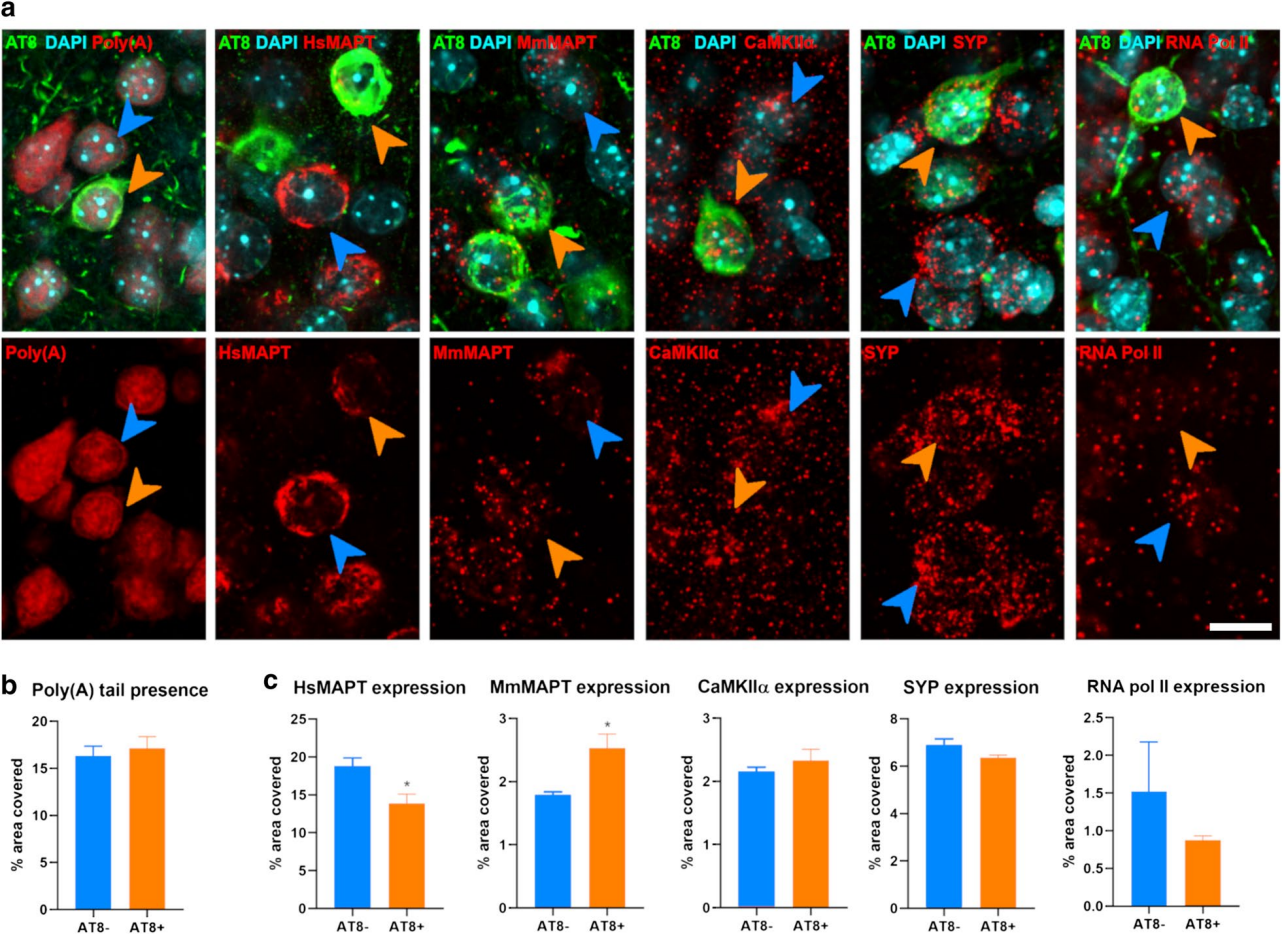
Transcript amount in NFT bearing neurons of old animals. **a** Single focal planes of neurons lacking (AT8-, blue arrowheads) or bearing NFT (AT8 + , orange arrowheads) for the total amount of mRNA (Poly(A) tail, left panel); and every gene analyzed. **b** Graphs summarizing the percentage of area covered with mRNAs (poly(A) tail). **c** Graphs summarizing the percentage of area covered with each probe targeting the genes of interest. Scale bar: 8 µm

In order to study if the general genetic transcription of AT8 positive neurons was affected, we first analyzed the amount of Poly(A) tail presence in those cells. This poly(A) is usually added at the end of pre-mRNAs as part of their processing, making their estimated quantity an accurate proxy for the total number of mRNAs. Young and old mice displayed no differences in this parameter when comparing AT8 positive to AT8 negative cells (*p* = 0.869 and *p* = 0.621 respectively; Figs. 2a, b and 3a, b). Likewise, to determine whether age had an effect on the general transcription, we compared the amount of Poly(A) signal present in neurons in young and old mice. Here, we did not find any significant changes within AT8- (*p* = 0.123) or AT8 + (*p* = 0.316) neurons (Additional file 1: Fig. S1).

### The expression of human MAPT decreases in tangle bearing neurons of old animals, whereas the expression of mouse MAPT increases

We next examined if the expression of individual key genes was altered. We chose to study the expression of human and mouse MAPT, as the transgene and intrinsic gene encoding tau, and CaMKIIα, SYP and RNA Pol II for their known relationships with AD [8, 9, 27]. We expected that human and mouse MAPT expression would not be changed by the presence of a phospho-tau protein inclusion. While in young animals this was the case for the transgenic expression of human MAPT (unchanged, *p* = 0.987), AT8 positive neurons showed a surprising trend towards an increase in the expression of mouse MAPT (*p* = 0.053, Figs. 2a, 2c). We then examined mouse and human MAPT expression in older mice (12-months-old), and these observations were even more striking. The expression of both human and mouse MAPT changed in neurons that overexpress the transgene and are AT8 positive. Human MAPT expression decreased in AT8 positive neurons (*p* = 0.014), while the expression of mouse MAPT was significantly increased (*p* = 0.018) in AT8 containing vs AT8 negative neurons in old animals (Fig. 3a, c).

### Expression of synaptophysin, CAMKIIα, and RNA Pol II in human tau overexpressing neurons that develop phospho-tau inclusions

In young animals, there were no differences in the expression of CaMKIIα (*p* = 0.425) and RNA Pol II (*p* = 0.261) in AT8 positive transgene expressing neurons. Surprisingly, in contrast to the results expected based on examination of human AD tissues, the expression of SYP was significantly increased (*p* = 6 × 10^−4^) in neurons that develop AT8 inclusions in young mice, potentially an early compensatory phenomenon (Fig. 2a, c). In older animals, the expression of CaMKIIα (*p* = 0.367), SYP (*p* = 0.126) and RNA Pol II (*p* = 0.369) was unaltered in AT8 positive neurons compared to AT8 negative tau transgene overexpressing neurons (Fig. 3a, c), again contrasting with expected results from human AD tissues.

### Neurons that are recipients of propagated tau show marked synaptic and metabolic effects

We next examined neurons that were apparent recipients of aggregated tau. The recipient neurons are defined as neurons that (by RNAScope) are negative for expression of human tau transgene, but contained a tau immunopositive aggregate as marked by AT8. Analyses analogous to those above were carried out, now comparing AT8 positive neurons that do not overexpress the transgene (HsMAPT-) vs those overexpressing it (HsMAPT +). Quantitatively, in both young and old animals ~ 20% of the total number of neurons that were AT8 positive were found to be recipients, i.e. not expressing the transgene but still containing phospho-tau (Fig. 1d). This suggests that a readily detectable minority of cortical neurons in the rTg4510 mouse expresses a small amount of transgene, and that, nonetheless, these transgene negative neurons contribute to the AT8 positive population of neurons.

**Fig. 4:**
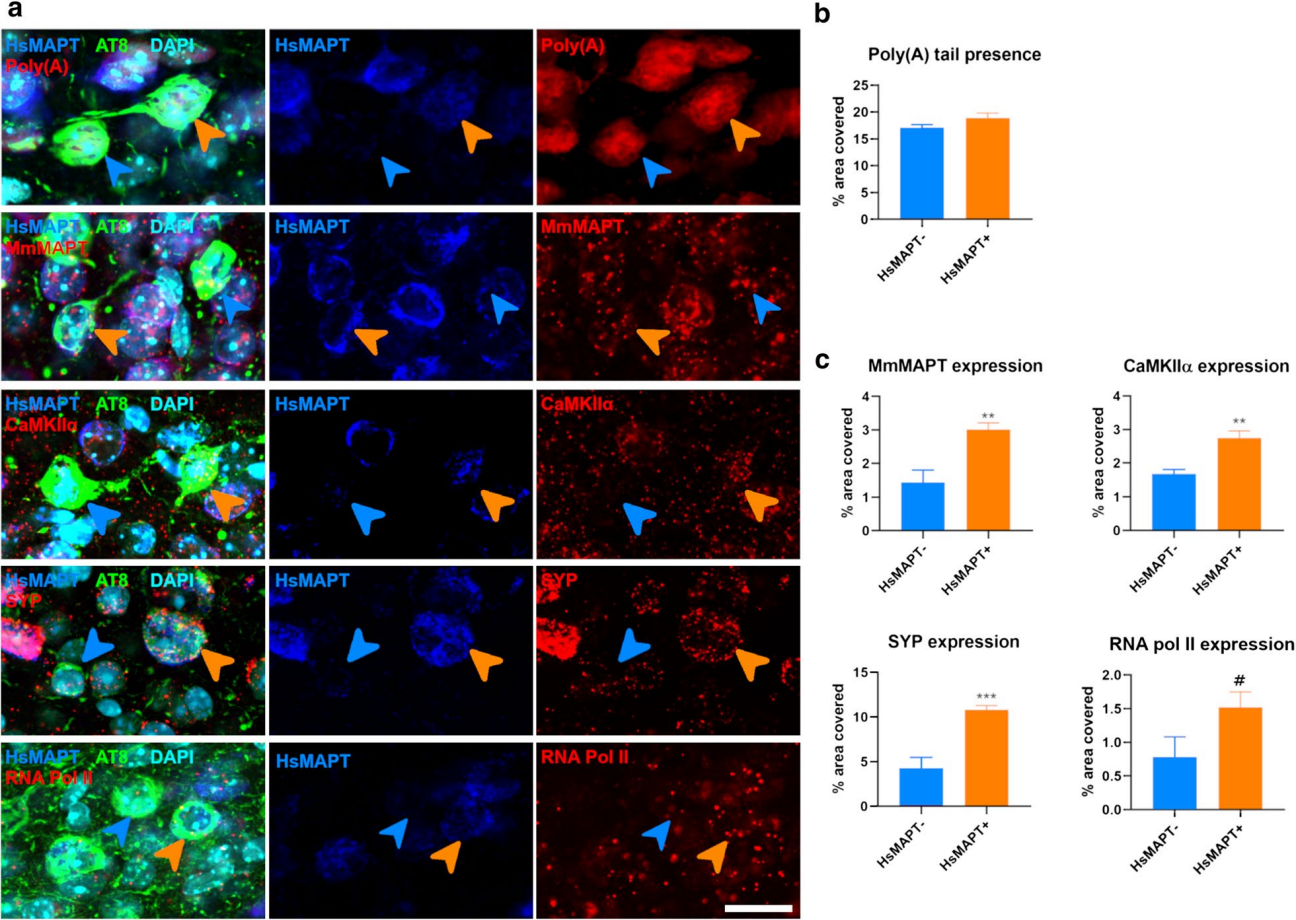
Transcript amount in NFT seeded neurons of young animals. **a** Single focal planes of seeded neurons (NFT bearing neurons lacking HsMAPT expression; HsMAPT- blue arrowheads) or NFT bearing, and not seeded, neurons (NFT bearing neurons expressing HsMAPT; HsMAPT + , orange arrowheads) for the total amount of mRNA (Poly(A) tail, left panel); and every gene analyzed. **b** Graphs summarizing the percentage of area covered with mRNAs (poly(A) tail). **c** Graphs summarizing the percentage of area covered with each probe targeting the genes of interest. Scale bar: 17 µm

Since recipient neurons (HsMAPT- AT8 +) could be identified, we were able to ask if they had a phenotype distinct from neurons that both overexpressed the transgene and became AT8 positive (HsMAPT + AT8 +). In this subpopulation, the amount of Poly(A) tail RNA was unchanged in young mice (*p* = 0.145, Fig. 4a, b) showing that the neurons remained broadly intact. Interestingly, however, in recipient neurons there was a trend towards a decrease in the expression of RNA Pol II (*p* = 0.097) and a significant decrease in the expression of mouse MAPT (*p* = 0.006), CaMKIIα (*p* = 0.002) and SYP (*p* = 6 × 10^−4^, Fig. 4a, c).

**Fig. 5:**
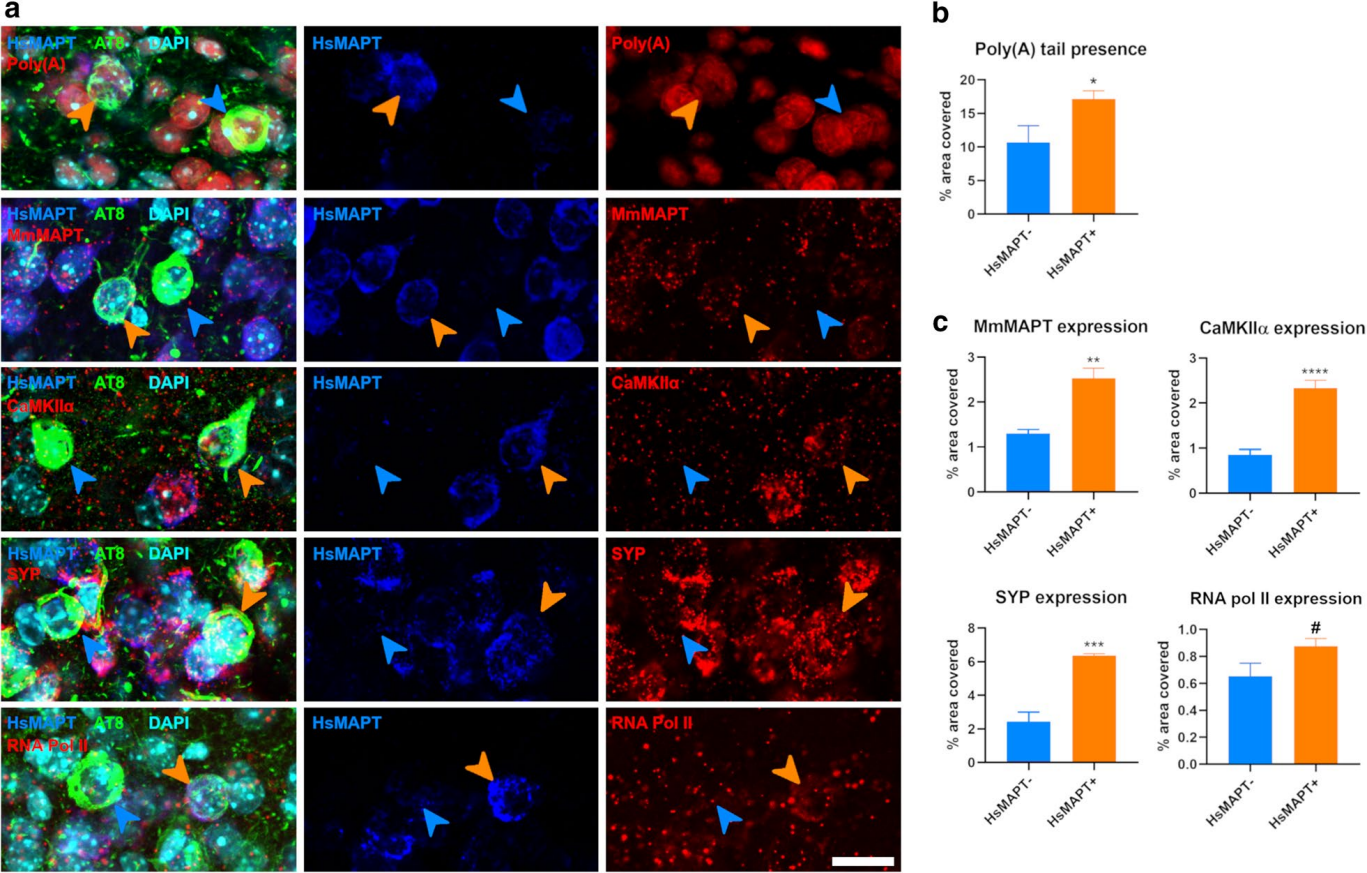
Transcript amount in NFT seeded neurons of old animals. **a** Single focal planes of seeded neurons (NFT bearing neurons lacking HsMAPT expression; HsMAPT- blue arrowheads) or NFT bearing, and not seeded, neurons (NFT bearing neurons expressing HsMAPT; HsMAPT + , orange arrowheads) for the total amount of mRNA (Poly(A) tail, left panel); and every gene analyzed. **b** Graphs summarizing the percentage of area covered with mRNAs (poly(A) tail). **c** Graphs summarizing the percentage of area covered with each probe targeting the genes of interest. Scale bar: 17 µm

These data were confirmed and extended in the recipient neurons observed in the second cohort of older animals. We found the amount of Poly(A) tail was significantly decreased in the recipient neurons (*p* = 0.049, Fig. 5a, b). More dramatic changes were seen in markers of metabolic and synaptic gene expression: we found recipient neurons showed a trend towards a decrease in the expression of RNA Pol II (*p* = 0.096) and a consistent and significant decrease in the expression of mouse MAPT (*p* = 0.002), CaMKIIα (*p* = 1 × 10^−5^) and SYP (*p* = 1 × 10^−4,^ Fig. 5a, c).

To ensure these changes were only occurring in cells receiving a tau aggregate, we next studied synaptophysin expression in AT8- neurons. We found there were no differences in this trait between HsMAPT- and HsMAPT + neurons in either young or old mice (Additional file 2: Fig. S2).

## Discussion

In this study, we examined the effect of human P301L transgene on gene expression in individual neurons in the rTg4510 mouse model of tauopathy, focusing on changes that had previously been shown to occur in human AD in synaptic and metabolic pathways [9]. The RNAScope approach allows us to identify individual neurons that both express the human tau transgene and develop AT8 positive aggregates, as well as a smaller subpopulation of neurons that develop an aggregate but do not overexpress the transgene (which we refer to as recipient neurons). Doing so, we were able to distinguish the effects of transgene overexpression and tau aggregation. We hypothesized that neurons that develop tau aggregation but without the consequences of overexpressing human tau, may better reflect what happens in AD brain, where of course aggregates occur without overexpression of a transgene.

Tau overexpression has multiple effects on gene expression measured by bulk analyses in the tg4510 mouse [10]. However, as the animals age there is dramatic neurodegeneration and glial activation [24]. A recent study has shown the transgene insertion site in rTg4510 mice disrupts the expression of other genes in this mouse strain, including *fgf14* [18], which may further complicate interpretation of bulk RNA analyses. Moreover, we have recently demonstrated that tau overexpression, in two separate transgenic lines, leads to diminished spontaneous activity of neural systems even in young animals [6]. It seems likely that many alterations in gene expression may be consequent to these potentially confounding issues of altered neural activity and neurodegeneration. Using RNAScope in situ hybridization techniques, we are able to address tau related alterations in the context of individual intact neurons, addressing at least some of the confounds noted above from bulk analyses. This approach also allowed us to focus on recipient cells that have the phenotype of having AT8 immunoreactivity, but not transgene overexpression, within the same environment. Of importance, the methodology inherently allows for a direct comparison between cells within the same animal. While this is experimentally advantageous in terms of reducing a variety of possible confounds, it diminishes the ability to definitively say the direction of change between two populations—i.e. if one went up or the other went down. In the context of a neurodegeneration model of tau overexpression, our underlying framework is that molecules that are known to be reduced in neurodegeneration, such as synaptophysin, would be expected to go “down” in a pathological context.

We find that the rTg4510 neurons that overexpress P301L tau do not necessarily mimic the gene expression changes observed in human AD –either before or after AT8 immunoreactive aggregate formation [8, 9, 27]. Moreover, the presence of AT8 positive tau aggregates do not affect general genetic expression through age, despite the fact that tau accumulation has been shown to be linked to impaired organelle transport and increased oxidative stress [28, 29]. We also show that AT8 aggregates do not impact the markers of synaptic or metabolic physiology that we examined compared to tau overexpressing only neurons that are not AT8 positive in the old cohort of animals.

We do observe some reciprocal relationships between human tau transgene overexpression (driven by a truncated form of the CAMKIIα promoter) and endogenous mouse tau, which remain largely unexplained. A recent report on the entorhinal cortex of rTg4510 animals, shows that mouse MAPT expression remained unaltered in diseased animals, while human MAPT increased drastically; a result consistent through ages of 2 to 8 months [10]. Our data adds a level of complexity to these results by showing alterations of these genes in NFT bearing neurons of 12 months old animals, with a decrease in the expression of human MAPT and an increase in mouse MAPT. Considering the sequences of human and mouse MAPT are homologous up to 87% [1, 2], this points to a potential auto-regulation of MAPT expression.

By contrast, excitingly, we find that recipient neurons that are not overexpressing tau mRNA but become AT8 positive seem to more closely recapitulate changes in markers of synaptic and metabolic physiology that are altered in human NFT bearing neurons, possibly in an age dependent fashion. In particular, we see significant decreases in a well-studied synaptic marker (synaptophysin), an enzyme critical for LTP phenomenon (CAMKIIα), and a trend towards a decrease in the enzyme responsible for transcribing most mRNAs (RNA Pol II). We even see a significant reduction of the general metabolic state of total poly(A) mRNA, altogether suggesting changes that match with observations in human tau aggregate bearing neurons in AD [8, 16]. We postulate that the difference between HsMAPT positive, AT8 positive and HsMAPT negative, AT8 positive cells may be in how the tau came to be in the cytoplasm, with the former due to cell autologous synthesis, and the latter due to uptake, with potential processing or conformational or post translational modification in the endosomal lysosomal compartments prior to release into the cytoplasm. Regardless of how the cellular consequences of AT8 aggregation differ in HsMAPT overexpressing cells compared to non tau overexpressing cells, these results are consistent with the hypothesis that tau transgene overexpression induces changes that only partially reflect the human biology that the mouse is trying to model, yet tau aggregation alone more closely reflects human tangle pathophysiology.

Tau seeding is well established to happen in vitro and in vivo [3, 7, 11, 13, 14, 17, 19, 25, 30], but to date no study has addressed how neurons respond to being the recipient of the transfer of proteopathic seeds. We show a significant decrease in the general transcript amount in seeded cells of old mice, and note that seeded cells have less expression of intrinsic mouse MAPT, CaMKIIα and SYP in both young and old animals. These results show that individual neurons that do not express the transgene but are exposed to the challenge of tau uptake, phosphorylation, and aggregation, show changes in synaptic and metabolic mRNAs that are reminiscent of what has been observed in human AD. This supports the hypothesis that, independent of tau transgene overexpression, tau aggregation of proteopathic seeds in recipient neurons negatively impacts neuronal well-being.

## Supplementary information

**Additional file 1.** Total transcript amount in neurons expressing HsMAPT transgene. Graphs summarizing the percentage of area covered by the Poly(A) tail in AT8- (left panel) and AT8 + (right panel) neurons.

**Additional file 2.** Synaptophysin expression in neurons without NFT. **a** Single focal planes of neurons not bearing an NFTs, lacking HsMAPT expression (HsMAPT-, blue arrowheads) or expressing HsMAPT (HsMAPT + , orange arrowheads). **b** Graphs summarizing the percentage of area covered with the synaptophysin probe. Scale bar: 12 µm.

## Data Availability

The datasets analyzed during the current study available from the corresponding author on reasonable request.
