# Supplementary figures and images for: Synaptic and metabolic gene expression alterations in neurons that are recipients of proteopathic tau seeds
Source: Acta Neuropathol Commun. 2020 Oct 19;8:168. doi: 10.1186/s40478-020-01049-7 (PMC7574323; doi:10.1186/s40478-020-01049-7)

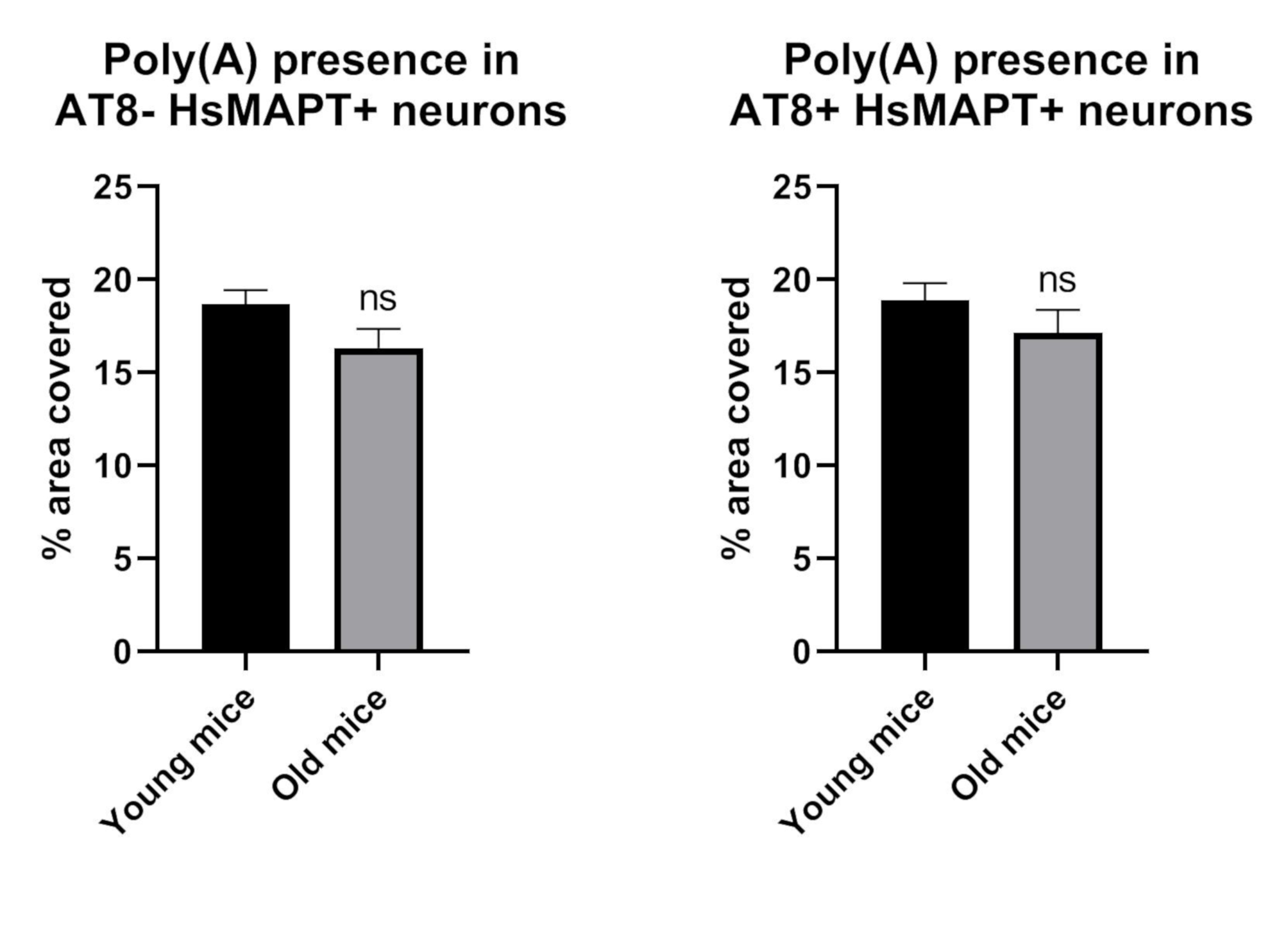

Supplement: Supplementary file 1 — Additional file 1. Total transcript amount in neurons expressing HsMAPT transgene. Graphs summarizing the percentage of area covered by the Poly(A) tail in AT8- (left panel) and AT8 + (right panel) neurons. [file 40478_2020_1049_MOESM1_ESM.tif]

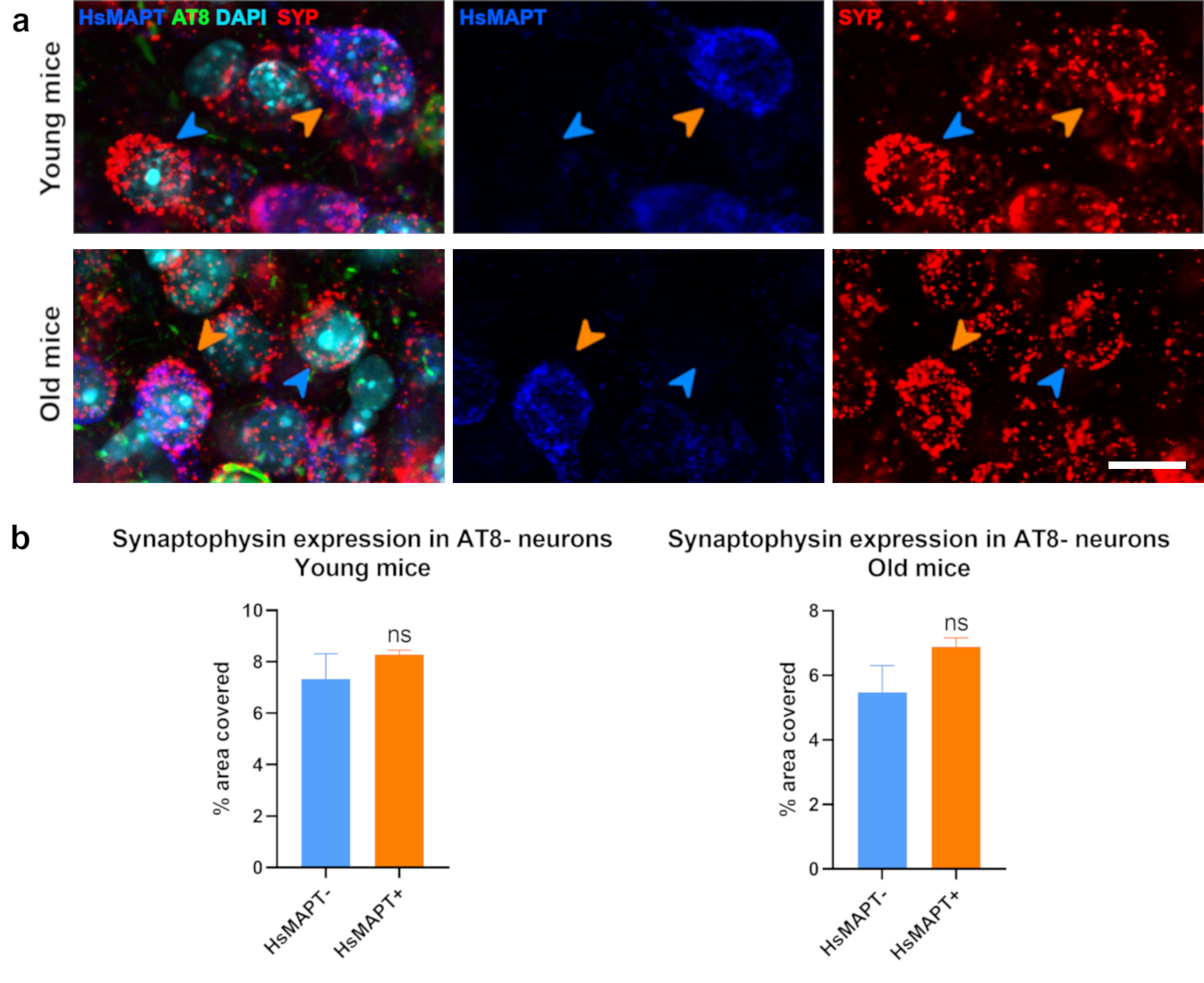

Supplement: Supplementary file 2 — Additional file 2. Synaptophysin expression in neurons without NFT. a Single focal planes of neurons not bearing an NFTs, lacking HsMAPT expression (HsMAPT-, blue arrowheads) or expressing HsMAPT (HsMAPT + , orange arrowheads). b Graphs summarizing the percentage of area covered with the synaptophysin probe. Scale bar: 12 µm. [file 40478_2020_1049_MOESM2_ESM.tif]
